# Omics-driven crop potassium use efficiency breeding

**DOI:** 10.3389/fpls.2022.1076193

**Published:** 2022-11-24

**Authors:** Bing He, Fengqin Hu, Hongyang Du, Junjie Cheng, Bingwen Pang, Xi Chen, Yuanda Lv

**Affiliations:** ^1^ Institute of Germplasm Resources and Biotechnology, Jiangsu Academy of Agricultural Sciences, Nanjing, China; ^2^ Excellence and Innovation Center, Jiangsu Academy of Agricultural Sciences, Nanjing, China; ^3^ Key Laboratory of Rice Genetic Breeding of Anhui Province, Rice Research Institute, Anhui Academy of Agricultural Science, Hefei, China; ^4^ School of Agronomy and Horticulture, Jiangsu Vocational College of Agriculture and Forestry, Jurong, China

**Keywords:** omics, KUE, crop, integration analysis, breeding

## Introduction

Potassium (K) is one of the essential mineral nutrients for plant growth and development, which plays a vital role in a wide range of physiological and biochemical processes in plants (Ragel et al., 2019). Plants must absorb plenty of potassium ions (K^+^) to maintain normal development and growth. However, many agricultural lands in the world lack K because of leakage loss and large-scale agricultural production (Zörb et al., 2014). K deficiency seriously limits plant growth and decreases crop yield and quality (Jordan-Meille and Pellerin, 2004; Gerardeaux et al., 2010; Wang et al., 2015; Hu et al., 2019).

Recent studies on K nutrition in plants mainly focus on K transport and signaling (Wang et al., 2021). Many K^+^ channels, transporters, and signaling pathways have been well studied, such as Shaker K^+^ channel AKT1, KUP/HAK/KT K^+^ transporter HAK5 and CBL-CIPK pathways (Xu et al., 2006; Véry et al., 2014). However, only a few of these genes have been used to improve crop KUE (Potassium use efficiency) through the transgenic approach. For example, the ectopic expression of the *WOX11* gene driven by the promoter of a low-K-enhanced K transporter improved rice tolerance to K deficiency (Chen et al., 2015). Applying K fertilizer is a common strategy for enhancing crop yield. However, the high input of K fertilizer and the low KUE increase production costs and have led to environmental problems. It is necessary to breed crop varieties tolerant to low K (Yang et al., 2003; Cao et al., 2007; Damon et al., 2007; Zhao et al., 2014; Song et al., 2018) to reduce K fertilizer and increase agricultural income and maintain the sustainable development of agriculture.

## Characteristics of crop varieties tolerant to low K

Usually, soil potassium deficiency inhibits crop growth and reduces crop quantity and quality. However, some crop varieties grow well and show a slight decrease in yields in the low-K field (Yang et al., 2004; Damon et al., 2007; Hu et al., 2016; Song et al., 2018). Varieties of this kind are recognized as low-K-tolerant varieties. Compared with low-K-sensitive varieties, low-K-tolerant varieties have a common characteristic: more robust K uptake capacity and higher K concentration. In soybeans, the total K accumulation per plant (mg/plant) of the low-K-tolerant variety is 2.36 times that of the low-K-sensitive variety under the K deficiency condition (Wang et al., 2012). In barley, the low-K-tolerant variety has a significantly higher shoot and root K concentration (mg/g dry weight) and K accumulation than the low-K-sensitive variety under the K deficiency condition (Ye et al., 2021). The same phenomena have been observed in other species like rice (Yang et al., 2004), maize (Cao et al., 2007), wheat (Zhao et al., 2020) and cotton (Tian et al., 2008). Furthermore, it has been observed that some low-K-tolerant varieties have more extraordinary K translocation ability than the low-K-sensitive varieties in the K deficiency condition. Low-K tolerant rice varieties preferentially distribute K to leaves at the booting and grain-filling stages to maintain stomatal conductance and ribulose diphosphate carboxylase (RuBPCase) activity in functioning leaves under low K, whereas low-K-sensitive varieties do not have this capacity (Yang et al., 2004). The efficient distribution of K to functioning leaves contributes to the relatively high grain yield in low-K-tolerant varieties at low K. Cotton has a high requirement for K, and lint yield and fiber quality could be damaged by K deficiency (Zhang et al., 2007). Jiang et al. (2008) found that low-K-sensitive varieties mainly transported K nutrition to vegetal organs (stems, branches and petioles), but low-K-tolerant varieties transported more K to the reproductive organs (bolls) that provide products. The efficient distribution of K to bolls probably help to maintain lint yield and fiber quality under K deficiency condition.

Therefore, plant K use efficiency is affected by the capability of K uptake and transportation and distribution of K. The low-K-tolerant crops should have better K uptake, transportation and distribution capability in low K soil, and all factors played important roles in crop yield and quality.

## High-throughput screening of crop varieties tolerant to low K

Tolerance to low K is a complex phenotype determined by genotype and environmental factors. To ensure the screening efficiency and accuracy, low-K-tolerant varieties should be screened through indoor and field experiments. Indoor experiments are usually performed in a growth chamber or greenhouse under hydroponic culture. It has the advantage of precise environmental control. Indoor experiments are usually carried out for screening at the seedling stage and thus could screen many varieties simultaneously. However, plant K nutrition is dynamic. Even a genotype probably shows different tolerance to low K at different growth stages. Tian et al. once screened five varieties that tolerant to low K at the seeding stage but sensitive to low K at the mature stage from 50 cotton varieties (Tian et al., 2008). Low-K-tolerance indicators in the seeding stage are commonly related to biomass and K uptakes, such as dry weight, leaf area, root length, number and volume, K accumulation amount per plant, and K content per unit dry weight. These indicators are not directly related to the economic productivity of crops like cotton, rice and canola (Woodend and Glass, 1993). Therefore indoor experiments are usually adopted for preliminary screening. Low-K-tolerant varieties selected through indoor experiments should be further confirmed in the field based on yield and quality indicators.

The K requirements are different for different crops. Some crops like soybean, potato, maize and cotton have relatively high requirements for K nutrition, whereas other crops like wheat and millet have a relatively low requirement for K nutrition. The K requirement of crops significantly differed at different growth stages. In rice, for instance, the K requirement maximizes from tillering period to the jointing stage. Therefore the treating concentration and treating time of K should be determined from crop physiological characteristics.

## Omics data-driven KUE breeding

**Figure 1:**
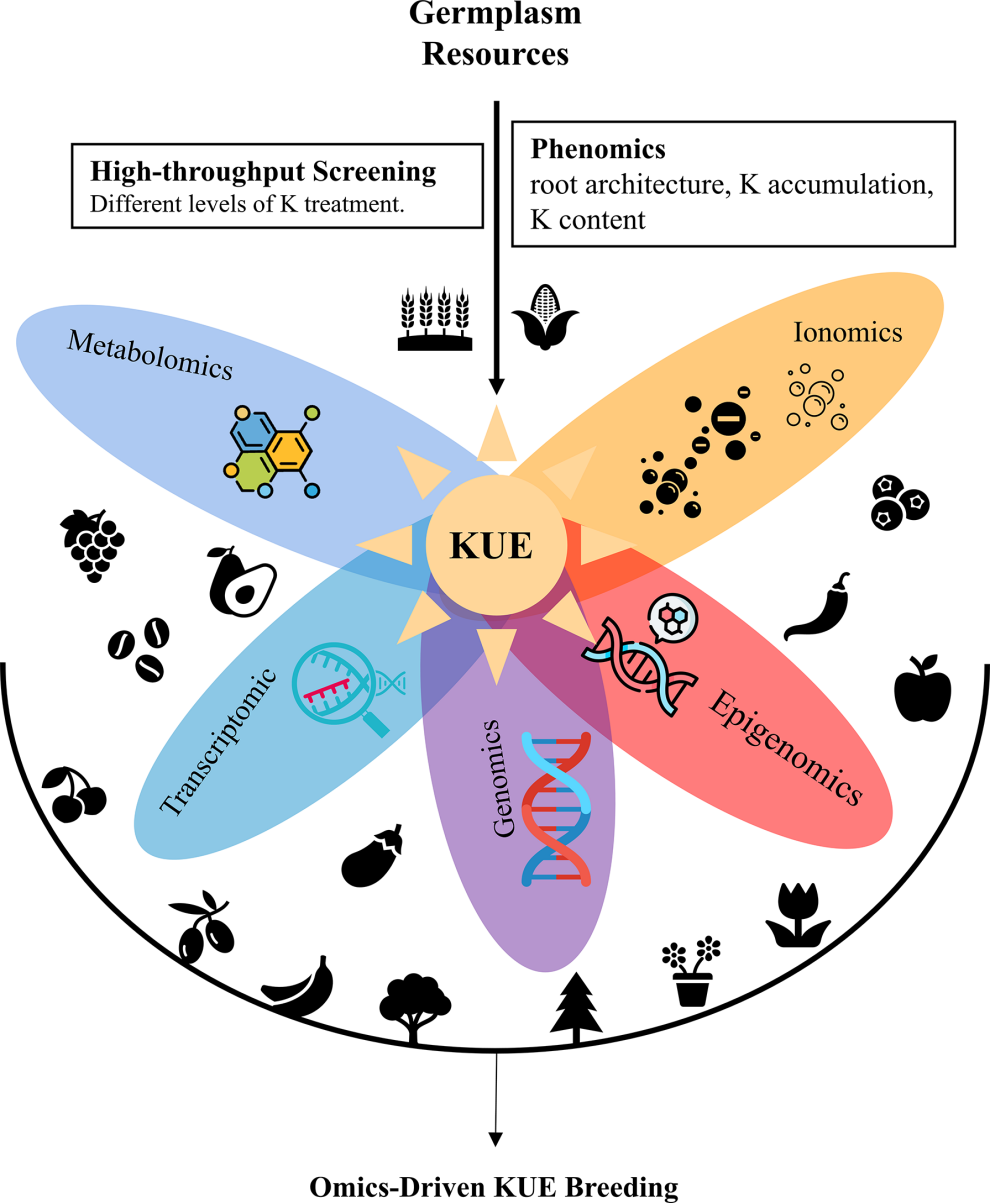
Integration of multi-omics technologies for accelerating crop KUE breeding.

Low-K-tolerant varieties can be directly used in agricultural production or applied in crop breeding as germplasm resources. The core of modern crop breeding is the introgression of genes responsible for good agronomic characteristics into the desirable genetic background. K-deficiency tolerance is a complex quantitative trait with strong interactions between genotype and the environment (Atienza et al., 2003; Prinzenberg et al., 2010). Many quantitative trait loci (QTLs) for low K tolerance have been identified *via* map-based cloning, and various molecular markers closely linked to these QTLs have been obtained (Wu et al., 1998; Prinzenberg et al., 2010; Zhao et al., 2014). With the help of marker-assisted selection (MAS) breeding, the crop breeding process has been accelerated. Nowadays, with a significant boom in omics technologies, omics-based interdisciplinarity further accelerates crop breeding (Li and Yan, 2020; Shen et al., 2022). Omics data such as genomics, proteomics, transcriptomics, metabolomics, phenomics, epigenomics allowed the understanding of physiological, biochemical, and molecular mechanisms underlining target traits and complex interactions between genes, proteins, and metabolites (**Figure 1**).

Genomics technology is widely exploited in genome diversification, evolutionary analysis, QTL mapping, and gene identification. High-quality reference genomes and vast genome-wide re-sequencing data greatly facilitate the identification of genes responsible for low-K tolerance *via* QTL mapping and genome-wide association study (GWAS). Wang et al. (2021) identified the candidate genes for QTLs that impact shoot dry weight at low K by whole-genome re-sequencing. Next-generation sequencing has helped construct high-resolution physical/genetic maps. Based on the high-resolution physical/genetic map, novel genomic loci controlling potassium use efficiency in bread wheat have been identified *via* GWAS (Safdar et al., 2020).

Transcriptomic data provides an insight into how genes are expressed under different K treatments, thus, have been extensively used to study genes, biological pathways and metabolic pathways that influence low K tolerance. Combining QTL mapping and transcriptome profiling helps to identify candidate genes for controlling low-K tolerance. Wang et al. (2021) investigated the variation in mRNA abundance of candidate genes between the two parental lines to assess whether any of these genes might contribute to K use efficiency. Transcriptome analyses of low K responsiveness have been performed in various crops, like rice, tomato, banana and maize (Fan et al., 2014; Shen et al., 2017; Zhang et al., 2017; Zhao et al., 2018; He et al., 2020; Ma et al., 2020). Genes coding transcription factors, transporters, kinases, oxidative stress proteins, and hormone signaling and glycometabolism-related genes are confirmed to be responsive to low-K signaling (Zhao et al., 2018). Gene regulation network roots under different potassium stress are constructed (He et al., 2020). Pathways related to jasmonic acid and reactive oxygen species production, Ca^2+^ and receptor-like kinase signaling, lignin biosynthesis and carbohydrate metabolism are significantly affected by K starvation (Fan et al., 2014; Shen et al., 2017).

Proteomics, metabolomics and ionomics studies about K use efficiency have been carried out in crops but are much less than transcriptomic studies. K deficiency could elicit complex proteomic alterations that refer to oxidative phosphorylation, plant-pathogen interactions, glycolysis/gluconeogenesis, sugar metabolism, and transport in stems (Li et al., 2019). Proteome-wide mapping of protein kinases indicates the potential role of CIPK23 and its substrates in regulating root architecture upon K starvation (Wang et al., 2020). Nkrumah et al. (2021) measure the variation in the ionome of tropical “metal crops” in response to soil K availability to study their K uptake ability at low K. The combined analysis of ionome and metabolome reveals the association between low K tolerance and drought tolerance in rapeseed (Zhu et al., 2020). A comprehensive transcriptome and ionome analysis showed the interaction among nitrogen (N), phosphorus (P) and K during the combined NPK starvation in sorghum (Zhu et al., 2020). Metabolomic and transcriptomic changes induced by K Deficiency provided new insight into the role of K in alleviating *Sarocladium oryzae* infection (Zhang et al., 2021). Leaf metabolome and proteome appear to be good predictors of carbon balance (Cui et al., 2019).

Phenomics and epigenomics studies about low K tolerance lag relatively behind. Epigenomics studies are mainly about non-coding RNAs and alternative mRNA splicing (AS). Long Non-coding RNAs sense environmental K concentrations and play regulatory roles in the K response network (Chen et al., 2022). AS modulation is independent of transcription regulation and plays a unique regulatory role in response to low potassium (He et al., 2021). Phenomics is the study of high-throughput phenotyping through crop development. However, phenomics data about KUE is relatively rare. Only Weksler et al. (2020) constructed a hyperspectral-physiological phenomics system to monitor and evaluate pepper response to varying levels of K fertilization.

## Prospects

K deficiency in farmland is a global problem. Improving KUE is a strategy to ensure higher crop productivity within sustainable environments. Low-K-tolerant varieties should have good K uptake and distribution capacities in low K soil to absorb sufficient K and prioritize the requirement for K of functioning organs. Generally, low-K-tolerant varieties are applied in crop breeding as germplasm resources. It is necessary to introgress low-K-tolerant traits into desirable genetic backgrounds or pyramid tolerance-related QTLs from different genetic resources. However, high-throughput screening technology of low-K tolerant varieties with efficiency and accuracy will still be a challenge. Omics data could reveal physiological, biochemical, and molecular mechanisms underlining low K tolerance and help identify QTLs determining KUE. With multi-omics development, we believe integrating these technologies will greatly boost the strategies of KUE breeding.

## Author contributions

YL and XC conceived and designed the study and revised the manuscript. BH wrote the manuscript. FH, HD, JC, and BP participated in data collection and analysis. All authors have read and approved the final manuscript.

## Funding

This work was supported by grants from Natural Science Foundation of Anhui Province (2108085QC108), National Natural Science Foundation of China (31601818), Natural Science Foundation of Jiangsu Province (BK20191224) and Project of Jiangsu Vocational College of Agriculture and Forest (2019kj005).

## Conflict of interest

The authors declare that the research was conducted in the absence of any commercial or financial relationships that could be construed as a potential conflict of interest.

## Publisher’s note

All claims expressed in this article are solely those of the authors and do not necessarily represent those of their affiliated organizations, or those of the publisher, the editors and the reviewers. Any product that may be evaluated in this article, or claim that may be made by its manufacturer, is not guaranteed or endorsed by the publisher.
